# Treatment of Corneal Neovascularization Using Anti-VEGF Bevacizumab

**DOI:** 10.1155/2014/178132

**Published:** 2014-03-23

**Authors:** Deli Krizova, Magdalena Vokrojova, Katerina Liehneova, Pavel Studeny

**Affiliations:** Ophthalmology Department, 3rd Medical Faculty of Charles University and University Hospital Kralovske Vinohrady, Srobarova 50, 10034 Prague, Czech Republic

## Abstract

*Purpose*. To evaluate antiangiogenic effect of local use of bevacizumab (anti-VEGF antibody) in patients with corneal neovascularization. *Methods*. Patients were divided into two groups. All patients suffered from some form of corneal neovascularization (NV). Patients in group A received 0.2–0.5 mL of bevacizumab solution subconjunctivally (concentration 25 mg/mL) in a single dose. Group A included 28 eyes from 27. Patients in group B applied bevacizumab eye drops twice daily (concentration 2.5 mg/mL) for two weeks. Group B included 38 eyes from 35 patients. We evaluated the number of corneal segments affected by NV, CDVA, and the incidence of complications and subjective complaints related to the treatment. The minimum follow-up period was six months. *Results*. By the 6-month follow-up, in group A the percentage reduction of the affected peripheral segments was 21.6% and of the central segments was 9.6%; in group B the percentage reduction of the central segments was 22.7% and of the central segments was 38.04%. In both groups we noticed a statistically significant reduction in the extent of NV. *Conclusion*. The use of bevacizumab seems to be an effective and safe method in the treatment of corneal neovascularization, either in the subconjunctival or topical application form.

## 1. Introduction

Corneal transparency is determined by many factors including avascularity. Since the year 1872, when Arnold demonstrated that the process of angiogenesis utilizes the striae of intracellular cement for neovascularization (NV) formation in the cornea [1], the results of new research examining the process of new vessel formation in the cornea have been published [2, 3]. Recent research has focused on understanding the mechanisms that keep the cornea avascular under homeostatic conditions and that provide an avascular healing process. These studies agree that corneal angiogenic privilege includes several active cascades and therefore is not a passive process [4–7].

Corneal NV is the pathological ingrowth of vessels to the cornea from the limbal vascular plexus. This process is the result of the chronic reduction of oxygen in the cornea. Physiologically oxygen is absorbed from the air. Other reasons for the formation of pathological vessels in cornea are corneal infections, trauma, and immunological processes. Corneal NV can be asymptomatic, but more often it results in severe visual disorders, and in some cases in practical blindness because of unfavorable corneal opacification. Common available therapy is limited to removing the primary cause of new vessel formation in the cornea, local application of corticosteroids, laser photocoagulation of bigger vessel strains, and corneal transplantation in extreme cases [3].

Many stimulators and inhibitors regulating the hemangiogenesis were isolated in vitro. Factors from the Vascular Endothelial Growth Factor (VEGF) family were shown to be the primary mediators of this process [8]. VEGF was originally identified as a stimulator of vascular permeability (called VPF (Vascular Permeability Factor)) but subsequently has been shown to be a mitogen and angiogenic factor, especially for endothelial cells. After VEGF isolation, further isolated factors from the VEGF family were named VEGF-B, VEGF-C, and VEGF-D. The original form is currently called VEGF-A. VEGF factors are a part of the VEGF/PDGF (platelet-derived growth factor) supergene family [6, 9, 10]. VEGF-A binds to the VEGFR-1 and VEGFR-2 receptors and its expression is strictly regulated [11]. Increased production of VEGF-A was observed in cases of hypoxia and during inflammation. Overproduction of VEGF-A was observed in tumor cell proliferation, similarly to corneal neovascularization formation. VEGF-A sustains several steps of angiogenesis including proteolytic activity, vascular endothelial cell proliferation, and migration and capillary lumen formation [10, 12]. The importance of VEGF-A in corneal angiogenesis was demonstrated experimentally on animal models by inhibiting NV after stromal application of an anti-VEGF-A antibody [13].

Bevacizumab (Avastin, Roche) is an antibody that acts against all isoforms of VEGF. This molecule inhibits the interactions between VEGF and its receptors, blocking any VEGF activity [14]. Bevacizumab is currently approved for the treatment of colorectal carcinoma, mamma carcinoma, non-small-cell lung carcinoma, and renal carcinoma. It is widely used “off label” for the treatment of choroidal neovascularization secondary to age related macular degeneration [15].

## 2. Materials and Methods

The case series was performed at the Ophthalmology Department of the 3rd Medical Faculty and University Hospital Kralovske Vinohrady, from December 2007 to June 2011. “Off label” use of bevacizumab for the treatment of corneal NV in both application forms was approved by the local Ethics Committee of University Hospital Kralovske Vinohrady. The tenets of the Declaration of Helsinki were followed and all patients gave their informed consent before enrolling.

The study was designed as a prospective, nonrandomized, and noncomparative case series. The patient groups included 66 eyes from 62 patients, 35 women and 27 men, aged from 19 to 84 years. All patients had a certain form of corneal NV. The aim of this study was to evaluate the antiangiogenic effect of the subconjunctival and topical application of anti-VEGF antibody bevacizumab on several diseases related to corneal neovascularization.

Patients were divided into groups A and B according to the means of application of bevacizumab. Patients in group A received a single-dose injection of 0.2–0.5 mL of bevacizumab subconjunctivally using an insulin syringe after applying topical anesthetic eye drops (oxybuprocaine 0.4%, tetracaine 0.1%) for 15 minutes. The concentration of the bevacizumab solution used for group A was 25 mg/mL. Group A included 28 eyes from 27 patients, 17 women and 10 men, with a mean age of 60 years (27–84). Patients in group B applied bevacizumab eye drops twice daily for two weeks. The concentration of the bevacizumab solution used for group B was 2.5 mg/mL. Group B included 38 eyes from 35 patients, 18 women and 17 men, with a mean age of 63.5 years (19–79). The minimum follow-up time was six months. The reason for dividing patients into 2 groups was the fact that we started using bevacizumab subconjunctivally the first year but continued to use eye drops topically in order to improve patient's comfort, while maintaining the same efficiency.

The patients were further divided into 4 subgroups according to the primary cause of corneal NV. Subgroup 1 included patients with pterygium; subgroup 2 included patients with NV on a donor disc after penetrating keratoplasty; subgroup 3 included patients with other ocular pathology (corneal leucoma after alkali burns, vascularized scars after corneal ulcers, NV after herpetic keratitis, and Stevens-Johnson syndrome or corneal NV of another etiology); and subgroup 4 included patients under preparation for high-risk penetrating keratoplasty (Table 1). Patients in subgroup 4 had a shorter follow-up time because of following surgery maximum 3 months after treatment. These patients were excluded from overall statistics.

Before initiation of the treatment, all patients underwent a standard slit lamp ophthalmologic examination of the anterior and posterior segments of the eye. Corrected distance visual acuity (CDVA) and intraocular pressure were measured. The follow-up examinations were performed in both groups the first, third, and sixth months after the treatments were started. Changes in corneal neovascularization were documented on digital photographs during each visit (SONY DXC-950P, 3CCD color video camera, Japan).

**Figure 1:**
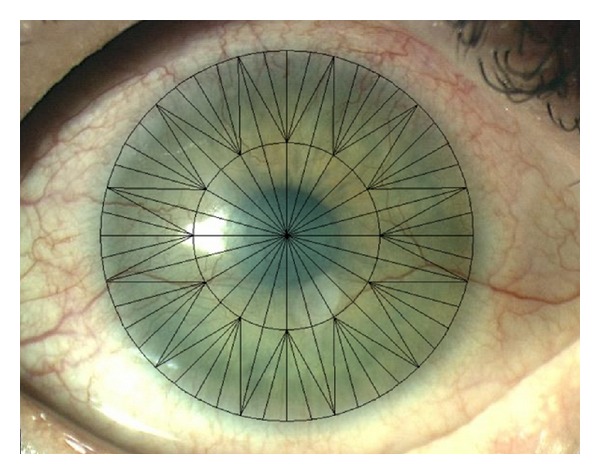
Pattern for the evaluation of the corneal neovascularization.

We used a special pattern to evaluate the extent of and change in corneal NV. These details were attached to the digital photograph of the cornea of each patient (Figure 1). The total diameter of the pattern was 12 mm with a central 6 mm zone. It consisted of 96 triangular segments of identical size, 72 peripheral segments (PS), and 24 central segments (CS). The extent of corneal NV was expressed in the number of segments containing blood-filled vessels. We evaluated the number of corneal segments affected by NV, CDVA, and the incidence of complications and subjective complaints related to the treatment. The evaluation of the masked photographs was done by a single doctor.

Each patient was treated with at least one application of the substance. Repeated treatment was not indicated before the first month following the first application of bevacizumab. We decided to repeat the treatment in the case of either a positive response to the first application, in order to achieve further regression of the NV, or a recurrence of corneal NV.

Using the SPSS Statistics program (version 19.0; SPSS, Inc., Chicago, IL, USA), a one-way ANOVA was performed for the purpose of statistical analysis. A *P* value below 0.05 was considered statistically significant.

## 3. Results 

### 3.1. Subconjunctival Injection of Bevacizumab: Group A

The mean number of affected peripheral segments and of central segments before the treatment that was initiated in group A was 30.37 (±23.40)—42.18% and 4.26 (±5.92)—17.75%, respectively. The mean corrected distance visual acuity (CDVA) before the treatment was 0.26 (±0.35). One month after the first bevacizumab subconjunctival injection, the mean number of affected peripheral segments and of central segments was reduced to 25.65 (±20.4)—35.63% (*P* = 0.003) and 3.42 (±5.44)—14.25% (*P* = 0.001), respectively. The mean number of repeated bevacizumab injections in group A was 2.07 (1–4). At six months after initiation of the treatment, the mean count of the peripheral segments and of the central segments was 23.81 (±17.80)—33.07% (*P* = 0.000) and 3.85 (±5.92)—16.04% (*P* = 0.000), respectively. The resulting mean CDVA was 0.28 (±0.36). The results of the separate subgroups are summarized in Table 2 and Figure 4.

The number of segments affected by corneal neovascularization at the one-month and six-month follow-ups compared with the number of segments before the treatment was statistically significantly lower.

Almost all of the patients tolerated the injection without any complaints, with the exception of patients who had experienced a chemical burn. Chemical-burn patients reported that the subconjunctival injection was very painful, even after the repeated application of anesthetic eye drops (oxybuprocaine 0.4%, tetracaine 0.1%).

We did not notice the progression of NV immediately after the injection in any of the patients in group A. We observed progression of NV in three eyes after initial regression. After further injections of bevacizumab, the NV reduced again in all three eyes. Picture 2 shows corneal NV in a patient treated with subconjunctival bevacizumab.

### 3.2. Topical Application of Bevacizumab: Group B

The mean number of affected peripheral segments and of central segments before the initiation of the treatment in group B was 27.94 (±20.29)—38.81% and 2.97 (±4.88)—12.38%, respectively. The mean corrected distance visual acuity (CDVA) before treatment was 0.55 (±0.42). One month after the topical application of the bevacizumab solution, the mean number of affected peripheral segments and of central segments reduced to 24.5 (±19.3)—34.07% (*P* = 0.000) and 1.97 (±3.59)—8.2% (*P* = 0.000), respectively. The mean number of repeated topical bevacizumab treatments in group B was 1.16 (1-2). At six months after initiation of the treatment, the mean count of peripheral segments and of central segments was 21.60 (±18.21)—30% (*P* = 0.000) and 1.84 (±3.79)—7.67% (*P* = 0.000), respectively. The resulting mean CDVA was 0.57 (±0.41). The results of the separate subgroups are summarized in Table 3 and Figure 5. Statistical analysis shows significant differences between the results at one-month and six-month follow-ups, compared with the number of affected segments before initiation of the treatment.

We noticed progression of NV immediately after the topical treatment in one patient with NV after a herpetic corneal ulcer. One month after discontinuing topical treatment, the NV regressed dramatically compared with the pretreatment level. In six eyes, we repeated the treatment three months after initiation because of repeated progression of corneal NV. All patients in subgroup B1 (pterygium) showed significant improvement in subjective complaints, that is, itching, a sense of the presence of a foreign body, cosmetically annoying redness of the eye.

## 4. Complications

We detected a systemic complication in only one patient from group A. It manifested itself 12 hours after the subconjunctival injection in overall weakness, headaches, and upper extremity paresthesia. These symptoms corrected themselves without any intervention, and we attributed them to a panic attack on behalf of the patient as a reaction to the treatment. Due to the patient's excellent local response to the treatment, we decided to continue the treatment of subconjuntival bevacizumab injections. The subsequent applications passed without complication. As for local complications, we observed tiny epithelial corneal defects in three patients in both groups A and B. The defects were completely healed after intensification of lubrication therapy. We detected hypersensitivity reactions in two patients from group B. In both cases, this reaction appeared on the third day after the initiation of the treatment. It manifested itself in eyelid edema and conjunctival hyperemia with a papillary reaction. Within two days, this condition was resolved in both cases by discontinuing the bevacizumab eye drops and replacing them with a treatment of fluorometholone acetate. We did not indicate any further treatment of bevacizumab eye drops in these two cases.

## 5. Discussion

The most current knowledge with regard to understanding the mechanism of ocular NV has led to the identification of new pharmacological goals. As VEGF plays a crucial role in the creation of corneal NV, its treatment with anti-VEGF antibodies seems to be the right method [7, 16, 17]. Several publications with this topic have already been published. Both subconjunctival injections [18, 19] and the topical use of bevacizumab [20–23] were experimentally used with promising results in treatment of herpetic keratitis [24, 25], recurrent pterygium [23, 26], corneal transplant rejection [19], and Stevens-Johnson syndrome [27]. The publications referred mostly to a small series of patients who had not undergone a uniform treatment scheme. Some authors reported the excellent effects of the anti-VEGF bevacizumab antibody in inhibiting and regressing corneal NV [18, 22, 24, 27]. No regression of corneal vascularization was observed in 2 studies involving cases of recurrent pterygium and corneal transplant rejection, after penetrating keratoplasty [28, 29]. Other studies proved some degree of regression of vessels from the affected cornea [19, 23, 30]. Complications were described in only a few studies, and always as superficial epithelopathy, tiny epithelial defects, or progressions of corneal thinning [22]. The reason for these adverse effects may be the fact that VEGF supports the growth of neural fibers and its blocking reduces the reparation of corneal nerves [31].

**Figure 2:**
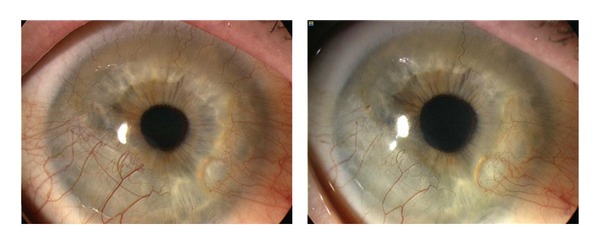
Representative case of corneal neovascularization treated with subconjunctival injection of bevacizumab. Patient was a 63-year-old female with chronic keratoconjunctivitis and rheumatoid arthritis. The baseline photograph shows circumferential (360 degrees) neovascularization (NV) of cornea (left). Six months after subconjunctival bevacizumab treatment, NV decreased significantly (right).

**Figure 3:**
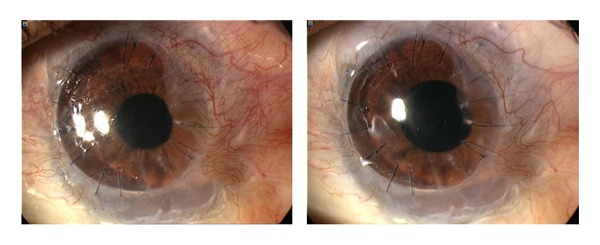
Representative case of corneal neovascularization treated with topical bevacizumab. Patient was a 19-year-old male who underwent penetrating keratoplasty combined with autologous limbal stem cell grafting for corneal leucoma after alkali burn. The baseline photograph shows active neovascularization (NV) reaching donor graft (left). Three months after topical bevacizumab treatment, NV decreased and is held on corneal graft border (right).

**Figure 4:**
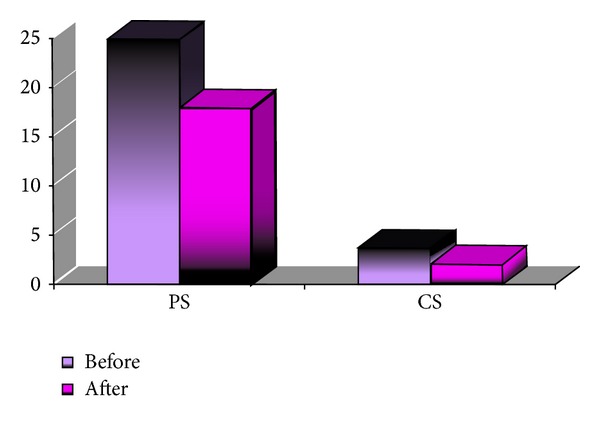
Comparison of affected peripheral (PS) and central (CS) segments before and after treatment in group A.

**Figure 5:**
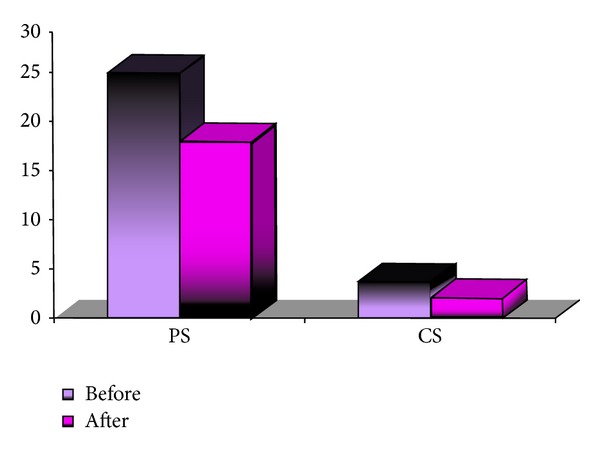
Comparison of affected peripheral (PS) and central (CS) segments before and after treatment in group B.

In our series we observed improvements immediately following the initiation of the treatment in the majority of patients and a stabilization of findings in all patients for a minimum of three months (Figures 2 and 3). Treatments using the anti-VEGF antibody bevacizumab provided statistically significant results. In all patients, we observed either an improvement in or stabilization of the corneal neovascularization. In both groups, mean visual acuity at the final follow-up had improved compared with the initial one. If there was any recurrence, we indicated another application of the bevacizumab treatment. With this regimen, we have been successful in keeping all monitored patients free of complaints for a long time. According to the particular cases with a longer follow-up (up to 15 months) and to the average number of retreatments in both study groups, the effect of the topical treatment seems to be more stable, without the need to repeat the treatment. Since the study was noncomparative, it is necessary to prove the hypothesis by means of a comparative study and on a larger group of patients.

We are convinced, based on the results of our study that the use of bevacizumab in the treatment of active corneal neovascularization could be beneficial. It may also be useful in high-risk keratoplasty, with regard to preoperative preparation and postoperative care. Thanks to the anti-VEGF antibodies, the blood vessels which can lead to corneal graft rejection are held beyond the corneal graft border. Our experience has shown that the use of bevacizumab seems to benefit the complex treatment of pterygium by reducing subjective complaints and delaying surgical intervention. While in some cases retreatment was necessary due to the temporary worsening of local findings, retreatment led to improvement.

However, treating corneal NV with the anti-VEGF antibody bevacizumab does have some limits. It is only a symptomatic treatment of corneal NV that does not cure the cause of the disorder and in some cases it is necessary to repeat the treatment to maintain its positive effect over a period of time. In addition, its effect on deep vascularization is lower in contrast to superficial and active vascularization, in which clear regression is observed.

Another possible limiting factor is the fact that anti-VEGF antibodies affect only one group of angiogenic agents. It is clear that the maintenance of the avascular cornea is an active process that requires an accurate balance between angiogenic and antiangiogenic mechanisms [7]. The use of other antiangiogenic factors or angiogenic inhibitors has been investigated using in vitro and experimental animal research. For example, the use of anti-PDGF antibodies seems to be an excellent supplementary therapy for anti-VEGF antibodies or the strong antiangiogenic factor PEDF [32].

## 6. Conclusion

The use of bevacizumab seems to be an effective and safe method in the treatment of corneal neovascularization, either in a subconjunctival or topical application form. The minimal incidence of complications and negative side effects promise the future evolution of the treatment as well as its adoption into broader clinical practice. Other clinical studies are necessary in order to evaluate the drug's efficacy, dosage, and safety in every case of corneal neovascularisation.

## Figures and Tables

**Table 1:** Distribution of patients into groups A and B and subgroups 1, 2, 3, and 4.

		Group A	Group B
1	Pterygium	6	12

2	NV after penetrating keratoplasty	8	6

3	Alkali burn	3	1
	Vascularized scar after corneal ulcer	3	2
	NV after herpetic keratitis	1	2
	Stevens-Johnson syndrome	2	1
	Other etiology	4	9

4	Preparation for penetrating keratoplasty	1	5

**Table 2:** Results of separate subgroups of group A before and 6 months after initiation of treatment; mean number of affected peripheral (PS) and central segments (CS) of neovascularization; corrected distance visual acuity (CDVA).

		Before treatment			6 months after treatment (1 month in group 4)			
		CDVA	PS	CS	CDVA	PS	CS	*P*
1	Pterygium (*n* = 6)	**0.85** (±0.21)	**8.5** (±5.12)	**0**	**0.9** (±0.18)	**6.66** (±4.49)	**0**	PS: 0.03
								CS: 0.2

2	NV after penetrating keratoplasty (*n* = 8)	**0.02** (±0.04)	**41.25** (±17.42)	**1.75** (±1.78)	**0.05** (±0.08)	**30.5** (±11.37)	**1.25** (±1.45)	PS: 0.001
								CS: 0.02

3	Other etiologies (*n* = 13)	**0.14** (±0.17)	**33.77** (±25.16)	**7.76** (±6.76)	**0.16** (±0.21)	**27.61** (±19.78)	**7.23** (±6.99)	PS: 0.000
								CS: 0.002

4	Preparation for penetrating keratoplasty (*n* = 1)	**0.0001**	**14**	**0**	**0.001**	**7**	**0**	

**Table 3:** Results of separate subgroups of group B before and six months after initiation of treatment; mean number of affected peripheral (PS) and central segments (CS) of neovascularization; corrected distance visual acuity (CDVA).

		** **			6 months after treatment (1 month in group 4)			
		CDVA	PS	CS	CDVA	PS	CS	*P*
1	Pterygium (*n* = 12)	**1.0** (±0.02)	**12.75** (±9.91)	**0.25** (±0.82)	**1.0** (±0.02)	**9.0** (±4.08)	**0**	PS: 0.003
								CS: 0.000

2	NV after penetrating keratoplasty (*n* = 6)	**0.06** (±0.04)	**35.5** (±20.13)	**0.83** (±1.21)	**0.12** (±0.13)	**28.0** (±23.12)	**0.33** (±0.74)	PS: 0.002
								CS: 0.000

3	Other etiologies (*n* = 15)	**0.58** (±0.32)	**37.0** (±21.49)	**3.4** (±4.01)	**0.59** (±0.32)	**29.53** (±18.73)	**2.53** (±3.98)	PS: 0.000
								CS: 0.000

4	Preparation for penetrating keratoplasty (*n* = 5)	**0.05** (±0.07)	**28.2** (±12.37)	**10.8** (±6.65)	**0.05** (±0.08)	**20.40** (±14.25)	**6.0** (±5.62)	PS: 0.02
								CS: 0.001
